# Patient-derived explants (PDEs) as a powerful preclinical platform for anti-cancer drug and biomarker discovery

**DOI:** 10.1038/s41416-019-0672-6

**Published:** 2020-01-02

**Authors:** Ian R. Powley, Meeta Patel, Gareth Miles, Howard Pringle, Lynne Howells, Anne Thomas, Catherine Kettleborough, Justin Bryans, Tim Hammonds, Marion MacFarlane, Catrin Pritchard

**Affiliations:** 1 Leicester Cancer Research Centre, University of Leicester, Clinical Sciences Building, Leicester, LE2 7LX UK; 2 LifeArc, Accelerator Building, Open Innovation Campus, Stevenage, SG1 2FX UK; 3 Cancer Research UK, Therapeutics Discovery Laboratories, London Bioscience Innovation Centre, 2 Royal College Street, London, NW1 0NH UK; 4 MRC Toxicology Unit, Hodgkin Building, Lancaster Road, Leicester, LE1 9HN UK

**Keywords:** Cancer models, Cancer models

## Abstract

Preclinical models that can accurately predict outcomes in the clinic are much sought after in the field of cancer drug discovery and development. Existing models such as organoids and patient-derived xenografts have many advantages, but they suffer from the drawback of not contextually preserving human tumour architecture. This is a particular problem for the preclinical testing of immunotherapies, as these agents require an intact tumour human-specific microenvironment for them to be effective. In this review, we explore the potential of patient-derived explants (PDEs) for fulfilling this need. PDEs involve the ex vivo culture of fragments of freshly resected human tumours that retain the histological features of original tumours. PDE methodology for anti-cancer drug testing has been in existence for many years, but the platform has not been widely adopted in translational research facilities, despite strong evidence for its clinical predictivity. By modifying PDE endpoint analysis to include the spatial profiling of key biomarkers by using multispectral imaging, we argue that PDEs offer many advantages, including the ability to correlate drug responses with tumour pathology, tumour heterogeneity and changes in the tumour microenvironment. As such, PDEs are a powerful model of choice for cancer drug and biomarker discovery programmes.

## Background

Cancer drug discovery and development is a challenging and expensive task, with estimates in 2015 suggesting that the development of a single new drug to the stage of regulatory approval costs ~$3 billion.^1,2^ Prior to approval, a new agent must pass through preclinical model systems and early-phase clinical studies in order to determine the mechanisms of action, probable efficacy and safety before assessment in randomised controlled trials (RCTs). Around 60% of new anti-cancer agents fail in RCTs,^2^ and the success rate from first-in-human to registration in oncology is ~5% – a figure that is much lower than that for many other diseases.^3,4^ The reason for this attrition, despite huge investment, is the subject of much speculation,^5–8^ but key factors include market pressures, challenges with the underlying science and the regulatory landscape.^9^

In terms of the scientific issues, the major causes of attrition have been determined to be a lack of efficacy (60% of failures), with safety issues accounting for a further 30%.^3^ Ways to address this attrition have been extensively discussed and include; building stronger evidence for the mechanism of action; establishing better preclinical models for efficacy testing; developing improved biomarkers to prove specific on-target response; earlier elimination of compounds with adverse toxicity; better designed proof-of-concept clinical trials; taking stronger decisions for discontinuation based on evidence at an earlier stage of the process. The assessment of all of these parameters is dependent on the availability of appropriate preclinical models that can be relied on to generate accurate, predictive data.^3,4^

Current approaches to studying the response of tumours to therapeutic agents comprise in vitro, in vivo and ex vivo models. Preclinical modelling has been compounded over the past few years by the increasing realisation that tumours are not as simple as was previously thought. There is now strong evidence for extensive inter- and intra-tumour heterogeneity both within and between human cancer samples^10,11^ and for a critical role of the tumour microenvironment (TME) in driving cancer hallmarks and in influencing drug responses.^12,13^ Indeed, the previous lack of appreciation for the diversity and complexity of human tumours might well have accounted for the attrition of many anti-cancer agents. An ideal ‘modern’ preclinical cancer model is one that can recapitulate both human tumour heterogeneity as well as the TME, and in the past 10 years or so, improvements have been made in available models to take into account this complexity.^14,15^ Despite this progress, there is a paucity of authentic preclinical models, particularly those that can predict responses to immune-checkpoint inhibitors,^16^ as these models require an intact TME. In this review, we provide an overview of existing models, such as organoids and xenografts, before outlining the potential use of explants derived from cancer patients, known as patient-derived explants (PDEs), as an ex vivo model of choice for use in the drug discovery pipeline.

## Current preclinical cancer models

**Fig. 1:**
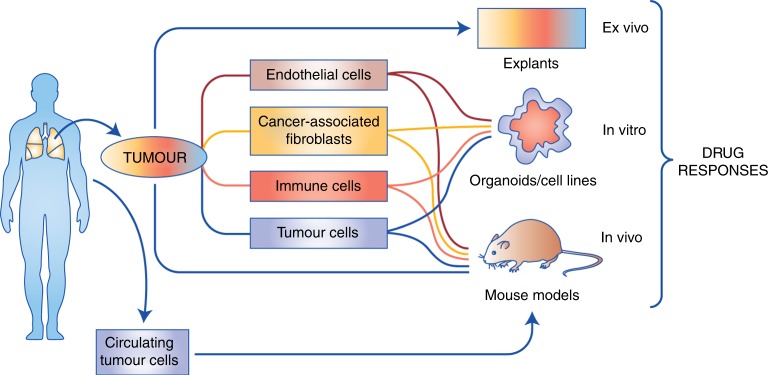
Available preclinical cancer models. In vitro and in vivo approaches generally involve deconstruction of the original tumours, and in some cases, reconstruction for subsequent assessment of drug responses, although patient-derived xenograft (PDX) models can preserve the integrity of original tumours following immediate transfer. Ex vivo approaches such as patient-derived explant (PDE) models assess drug responses directly in tumour samples obtained fresh from surgery without deconstruction/reconstruction. The schematic shows the cell types available for derivation and use in each model system.

Preclinical models that are currently used as drug-testing platforms fall into three broad categories: in vitro, in vivo and ex vivo. In vitro and in vivo models rely on deconstruction of the original human tumours, and in some cases, subsequent reconstruction with combinations of select cell types and assessment of drug responses. By contrast, ex vivo models facilitate drug testing of intact human tumours, without any requirement for deconstruction or reconstruction.^17^ A summary of available model systems is shown in Fig. 1.

### In vitro models

The starting point for preclinical research is usually two-dimensional (2D) cancer cell lines, which are easy to manipulate and generate data rapidly. Many 2D cell lines that have been deconstructed from human tumours and cultured on plastic under in vitro conditions are available to the cancer research community.^18^ These cell lines are valuable reagents for generating important biological and mechanistic understanding of cancer targets, as well as for the testing of anti-cancer agents. Indeed, with regard to drug discovery, the most significant development has been the compilation of the Genomics of Drug Sensitivity in Cancer (GDSC) database (www.cancerRxgene.org) – a large public resource that provides information on drug sensitivity and resistance in cancer cell lines combined with molecular analysis of drug response.^19–21^ This database has been constructed from an ongoing high-throughput screen of  >1000 cell lines by the Cancer Genome Project at the Sanger Institute, UK and the Centre for Molecular Therapeutics at Massachusetts General Hospital, USA, and is an invaluable resource for the community by providing a first assessment of the molecular features that influence drug responses in cancer cells. However, the caveat that all data are generated in 2D cell lines, which lack a microenvironment and the heterogeneity of tumour cells observed in vivo, must be borne in mind when interpreting drug responses.

An advance on the 2D cell line is the 3D model, also known as an organoid.^17,22^ Organoids are generated by the deconstruction of fresh human tumours and culture of tumour-derived cells in a semisolid extracellular matrix under defined culture media conditions. In most cases, studies have been conducted on organoid cultures derived from primary tumours,^23,24^ although a few examples of successful cultures derived from metastatic cancer sites exist.^25–27^ Patient-derived organoids have the advantage that tumour cells are grown in 3D, which is thought to better recapitulate growth in human tumours. Advances in tissue engineering technologies, such as new biomaterials and microfluidics, have facilitated improved culture quality and reproducibility in some circumstances.^28–30^ It is also possible to incorporate heterologous, stroma-derived cells, such as cancer-associated fibroblasts, immune cells and endothelial cells^31^ into 3D organoid cultures; some of these cells might be derived from the same patient, thereby effectively mimicking tumour growth in vivo.^32^ A co-clinical trial compared the responses of organoids with anti-cancer agents with the responses of the same patients to matched anti-cancer agents in clinical trials and showed recapitulation of the responses in the clinic.^27^ Several organoid cancer biobanks now exist for testing novel anti-cancer drugs, and organoids from healthy tissues are also available for toxicity testing.^23,33,34^

### In vivo models

In vivo models mostly refer to mouse models for cancer and have been extensively reviewed elsewhere.^35,36^ Mouse models comprise xenograft, spontaneous or genetically modified models, with xenograft models being most widely utilised in the drug discovery pipeline for assessing drug safety, pharmacokinetic and pharmacodynamic properties as well as drug efficacy. Mouse xenografts can be generated by the transfer of human cancer cell lines, organoids or fresh tumours, generally into immunocompromised hosts, such as athymic nude mice or mice with severe combined immunodeficiency (SCID). The potential to propagate circulating tumour cells (CTCs) in mice has also been demonstrated, as shown by the generation of CDX (circulating tumour cells)-derived xenografts from CTCs obtained from patients with small-cell lung cancer.^37^ An alternative is to use syngeneic or allograft mouse tumour systems in which mouse tumour tissues or cell lines are implanted into animals from the same genetic background, thus retaining an intact immune system.^38^

Patient-derived xenograft (PDX) models, in which fragments of fresh tumours are transplanted and propagated in immunodeficient mice, have received considerable interest and have been utilised for both basic and translational cancer research, biomarker discovery, personalised drug screening and understanding the mechanisms of drug resistance.^24,39,40^ Unlike cell line or organoid xenografts, PDX models have been suggested to retain the architecture and morphology of the original transplanted tumours,^41^ even after serial propagation, although there is some evidence that clonal selection occurs over time,^42^ so that the heterogeneity of the tumours may be lost.

An advantage of PDX models is that the propagated tumours can be excised and frozen, re-transplanted to new recipient mice and expanded at a later date.^42^ This also allows for continuous expansion of the original tumour between multiple recipient mice. As a result, collections of PDX models are available through commercial or not-for-profit organisations, which allow the exchange of well-characterised PDX models for multiple solid tumour types.^43,44^ PDX models also have the potential to be used to make treatment decisions for patients using ‘avatars’ generated from patient-matched surgically resected tumours. A particular example of this has been described for pancreatic cancer, in which PDX models were used to identify personalised treatment regimens, leading to improved responses in patients.^44,45^ However, there are hurdles to the use of PDX models as avatars, an important factor being the fact that PDX generation does not work for all tumours, with implantation rates reported as being anywhere between 23 and 75%.^46^ There is also a time delay associated with generating data from avatars, which compromise immediate patient treatment.

An important feature of any preclinical model is the ability to recapitulate the complexity of human tumours, including the TME. One of the problems with PDX models is that they make use of immunodeficient hosts and, although the tumour is not deconstructed prior to transplantation, infiltration of the TME by murine stroma occurs with serial propagation. This is a particular issue in the context of testing immunotherapies that work against human-specific immune checkpoints, and therefore, there has been particular interest in developing methods for human immune reconstitution within PDX models.^47^ Several approaches are under investigation to generate so-called ‘humanised mice’, including the use of immunocompromised mice harbouring human haematopoietic stem cells as recipients^48^ or reconstitution with autologous peripheral blood mononuclear cells or tumour-infiltrating lymphocytes (TILs).^49^ An alternative method is to genetically manipulate the host to express relevant human genes.^50,51^ For example, NSG-SGM3 mice represent immunodeficient mice engineered to express three human transgenes: stem cell factor, granulocyte/macrophage colony-stimulating factor and interleukin-3, thereby allowing long-term engraftment of human samples with expansion of human immune cells.^50^

### Ex vivo models

Ex vivo models mostly represent so-called ‘explant’ models, whereby fresh, surgically resected tumour is used immediately for drug studies without deconstruction of the tumour. Tumour-derived explants have been used since the 1950s, but despite some successes, they have not been widely accepted in the drug development pipeline to date. Below is a summary of the PDE platform, with a focus on the advantages this platform offers compared with the aforementioned in vivo and in vitro approaches.

## PDE cultures

### A historical perspective

**Fig. 2:**
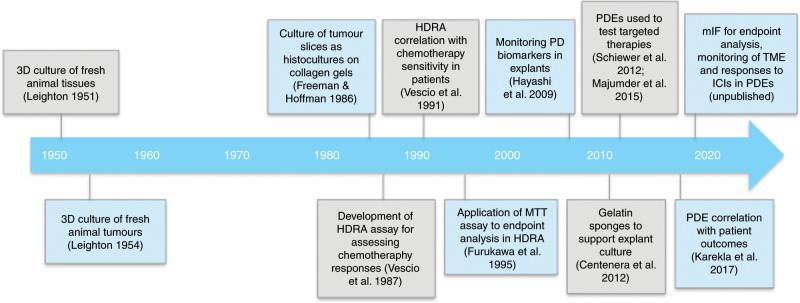
Timeline indicating the historical development of patient-derived explant cultures. HDRA histoculture drug response assay, PD pharmacodynamic, MTT 3-(4,5-dimethylthiazol-2-yl)-2,5-diphenyltetrazolium bromide, PDEs patient-derived explants, mIF multi-immunofluorescence, TME tumour microenvironment, ICI immune-checkpoint inhibitor.

A timeline of the development of PDE culture systems is illustrated in Fig. 2. Three-dimensional culture systems from fresh animal tissues were first developed in the early 1950s and were based on Leighton’s theory (1951) that ‘spatial conditions encountered by animal cells is one of dense three-dimensional masses of cells with limits to migration, and with gradients of both diffusion of metabolites and of morphologic maturation’.^52,53^ Early observational studies using sponge-matrix animal histocultures showed maintenance of the in vivo architecture of advanced tumours from adenocarcinomas, ovarian cancers, teratomas, malignant melanomas, reticulum cell sarcomas, lymphosarcoma, ovarian papillary adenocarcinomas and hepatomas.^54,55^

The first human tumour histoculture studies were pioneered by Robert Hoffman et al. and involved the generation of tissue slices from tumours obtained directly from surgery that were then cultured for extended periods of time using specialised collagen gels.^56^ This approach ensured that the stromal component within the TME remained intact. Cells were found to maintain properties of their in vivo state, including spatial organisation and differentiated cell function, and the growth of multiple cell types within a single tumour was observed.^56^

Histocultures were soon adapted for use in drug studies for assessing chemotherapy responses and were subsequently termed the Histoculture Drug Response Assay (HDRA).^57,58^ HDRA studies reported an 86% accuracy in predicting drug resistance for stomach and colon cancer,^58^ and a correlative HDRA clinical trial showed a 92.1% correlation rate for predicting drug resistance to clinical drug sensitivity in advanced gastric and colorectal cancers.^59^ A number of studies applied the HDRA to the prediction of drug response in non-small-cell lung cancer (NSCLC) and also showed good predictability rates.^60–64^

Different ex vivo culture methods have been developed over time by different research groups, such that there are many variables between reported methods.^65^ For example, explants can be cultured using the submersion method, in which tissue is completely submerged in media in tissue culture dishes; by the grid method, in which tissue is kept in contact with media through a matrix or membrane supported by a plastic or metal grid; by the sponge method, in which tissue is cultured on a gelatine or collagen sponge that sits in media. Gelatine sponges have been pioneered by Butler et al. and are thought to be an advance as they can facilitate distribution of media, nutrients and drugs through the explants without having to submerge the tissue in media.^66,67^ Tissue may also be maintained as fragments or processed for generation of tissue slices of uniform 200–300 μm sections, which are then cultured. The latter approach may not only facilitate drug diffusion, but may also result in loss of some tissue integrity due to the increased set-up time.

A variety of endpoint assays have also been applied to the explant approach to quantitate responses to various chemotherapy regimens. Most studies involved enzymatic digestion of tumours, followed by assessment of cell viability or cytotoxicity using the MTT (3-(6)-2,5-diphenyltetrazolium bromide) assay, the lactate dehydrogenase (LDH) assay or the use of Alamar Blue™.^59,60,68–70^ The MTT endpoint assay was most widely adopted for clinical correlation, and was used to develop an empirical value for HDRA responses to chemotherapy regimens.^59–61,71,72^ Typically, mean data from four treated tumour culture wells were determined by calculating the spectrophotometric absorbance of MTT solution for both drug-treated and control tissue and adjusted for tumour weight, generating an inhibition rate as follows: inhibition rate (%) = (1−mean OD/weight of treated well/mean OD/weight of control well) × 100.

Other studies used in situ approaches to assess drug responses, including autoradiographical assessment of nuclear incorporation of ^3^H-thymidine or immunohistochemistry for cell death or proliferation markers.^56–58,68^ Modified versions of the HDRA also included assessment of specific immunohistochemical biomarkers, including those for phosphatidylinositol 3-kinase (PI3K)-AKT, pro-apoptotic regulators and novel drug-resistance genes such as class III β-tubulin.^64,70,73^ Quantitative RT-qPCR has also been employed in more recent studies to screen for personalised biomarkers.^69,70,73,74^

Despite the evident success of the HDRA and other explant platforms in predicting patient responses to chemotherapy during the 1990s and 2000s, the platform has not been widely used since that time. The reasons for this are not entirely clear but may be related to the burgeoning use of cell line, organoid and PDX model systems, all of which can be propagated for longer periods of time. However, with the growing appreciation of the importance of the tumour microenvironment and tumour heterogeneity in tumour evolution and drug response, a more contextualised model system has been sought, hence the rejuvenation of the explant approach.

### Recent advances in PDE-based platforms

Majumder and colleagues have created the CANScript platform, in which freshly resected tumours are thinly sliced and cultured in tissue culture wells coated with a tumour matrix support, comprising tumour–stromal matrix proteins (TMP), in the presence of autologous serum derived from the same patient, creating a tumour ecosystem.^75,76^ They have used this platform with head and neck squamous cell carcinomas (HNSCC) and colorectal carcinomas (CRC) and evaluated responses to cytotoxic and targeted therapies by immunohistochemical assessment of cell death or proliferation biomarkers as well as pharmacodynamic biomarkers.^77,78^ Strong correlations between CANScript responses to cytotoxic drug regimens of docetaxel, cisplatin and 5-fluorouracil (TPF) and the responses of PDX models derived from the same human tumour samples to the same chemotherapies were observed. Furthermore, following stratification of HNSCCs for *KRAS* mutation status, correlations between the CANScript and PDX responses were observed upon treatment with the epidermal growth factor receptor (EGFR) inhibitor cetuximab. The platform was also able to predict clinical non-response, partial response or complete response in the same patients treated with TPF.^75^ On the back of these results with CANScript, an India–USA company has been formed, Mitra Biotech (www.mitrabiotech.com), which has the aim of personalising cancer treatment using PDEs.

**Fig. 3:**
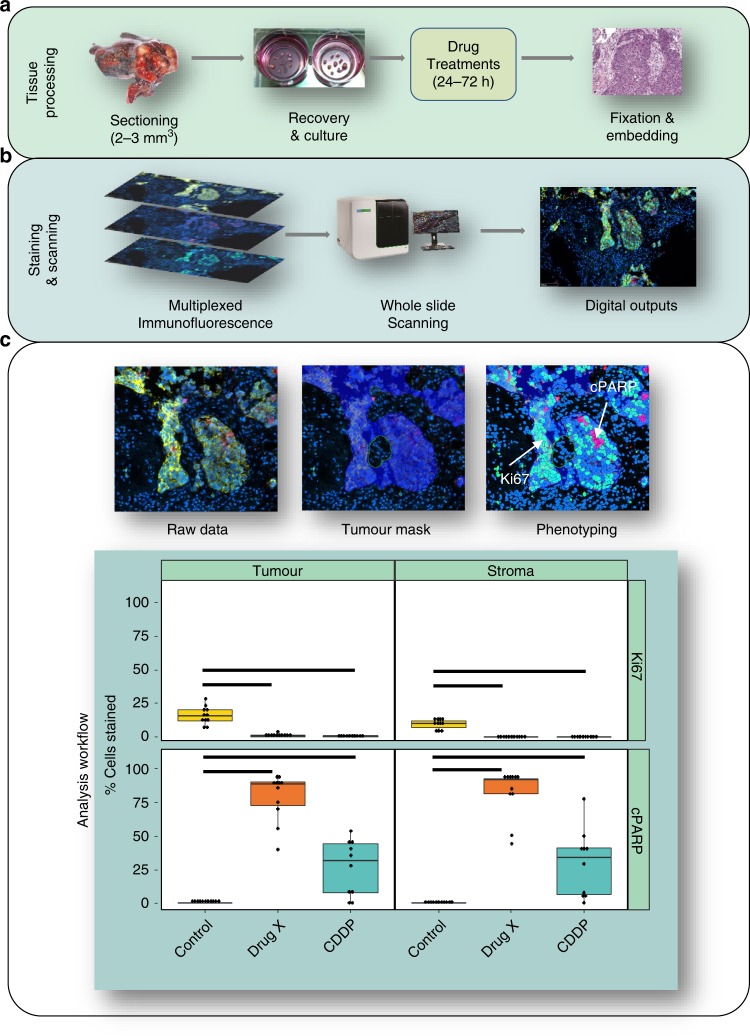
Workflow for PDE culture showing multiplexed immunofluorescence outputs and assessment of drug responses in PDEs. **a** shows the method for tissue processing, **b** shows the staining and scanning method and **c** shows the analysis workflow. In **c**, the image on the top left shows merged multi-immunofluorescence (mIF) staining of a non-small-cell lung cancer (NSCLC) explant with Ki67, cPARP, pan-cytokeratin and DAPI. The application of the tumour mask (middle) and digitisation of the image (right) allows segregation of staining in the tumour and stroma. The graphs at the bottom depict four quadrants showing % proliferation (Ki67) and % cell death (cPARP) in the stroma and tumour for the NSCLC PDEs treated with vehicle control, cisplatin (CDDP) or experimental Drug X. The PDEs were more responsive to Drug X when compared with cisplatin in both tumour and stroma tissue. Each point represents one PDE with boxplots displaying the first and third quartile (hinges), and median (centre line) with error bars representing the range no further than 1.5× IQR (interquartile range). Significance bars indicate *P* < 0.05 according to the Kruskal–Wallis test. The findings in this Figure are the authors’ unpublished original data.

An additional PDE platform developed by our own group uses an alternative approach, in which fresh NSCLC tumours are fragmented into 2–3 mm^3^ explants and cultured on membranes at the air–liquid interface (Fig. 3).^79^ PDE responses to the chemotherapy drug cisplatin showed a significant relationship with patient outcome, regardless of tumour stage.^79^ In this study, endpoint analysis was performed by immunohistochemical assessment and quantitation of Ki67 staining as a proliferation marker and cleaved poly-ADP ribose polymerase PARP (cPARP) as a cell death marker, thus allowing spatial evaluation of drug responses. The same PDE approach has also been applied to breast cancer,^80^ CRC^81^ and mesothelioma.^82,83^ In the breast cancer study, PDE responses to the targeted therapy TRAIL were found to be more consistent with clinical trial data than 2D tumour model systems.^80,84^

The gelatine-sponge approach has proved successful for the culture of explants derived from breast and prostate tumours,^85^ and has been applied to the testing of novel anti-cancer agents,^66,67,86,87^ development of biomarkers^87,88^ and for monitoring changes in the tumour microenvironment.^89^ In these studies, the PDE approach contributed important information to show the effect of the PARP inhibitor ABT888 in suppressing tumour cell proliferation in human prostate cancers^86^ and the effect of progesterone in inhibiting the proliferation of the oestrogen-mediated growth of ERα + breast cancers.^87^ In a separate study, Mariel et al. also examined changes in the TME in breast cancer explants in response to paclitaxel treatment and demonstrated decreased natural killer (NK) cell infiltration in resistant samples.^69^

### Endpoint analysis

In addition to developments in the PDE platform itself, there have been key technical developments in endpoint analysis. Following drug treatment, two options are available for evaluation of biomarkers that measure drug response. Either the PDE can be homogenised, as is the case with the HDRA assay, or it can be retained intact and processed for spatial biomarker analysis. Once a PDE is homogenised, protein, DNA, RNA or metabolites can be isolated and measured using a variety of different approaches, such as mass spectrometry and transcriptomic, genomic or metabolomic profiling. New developments such as single-cell RNA sequencing also allow the longitudinal characterisation of changes within tumour cells themselves or in the TME following drug treatment. However, an advantage of spatial profiling is that it allows interrogation of the relationship between drug responses, tumour pathology and tumour heterogeneity within the same sample.

For spatial profiling, PDEs are processed for formalin fixation, paraffin embedding (FFPE) prior to the analysis of histological sections using immunostaining approaches. New technological developments in multispectral imaging have made it possible to not only study tumour cells but also differentiate cell types in the TME, including immune cells. Identification of immune-cell subsets within tissue often requires multiple markers for accurate phenotyping; consequently, traditional immunohistochemistochemistry (IHC) techniques, which typically only stain for one protein/marker at a time, are inadequate for this purpose. Multiple methods now exist for overcoming this limitation, each of which has its own pros and cons. The most conventional is to stain serial sections individually by IHC using different markers, image them and align the sections in silico. Whilst relatively inexpensive, the major drawback of this method is its failure to acquire co-localisation and co-expression information at the single-cell level, therefore making it unsatisfactory for immune-cell phenotyping.

An alternative method is to use tyramide signal amplification (TSA). This is a comparable technique to traditional IHC, whereby primary antibodies recognising the epitope of interest are applied to the sections followed by horseradish peroxidase (HRP)-conjugated secondary antibodies and a final signal amplification step achieved via the covalent deposition of tyramide-conjugated fluorophores.^90–92^ This final covalent linkage makes this method particularly suited to multiplexed imaging, as each primary/secondary antibody complex can be stripped from the slide, typically by microwaving, and subsequent antibody–fluorophore pairs added sequentially. The additional benefit of this method is that as the fluorophore is covalently linked but the primary antibody removed, multiple primary antibodies of the same species can be used on one section.^93^ This methodology, in combination with new whole-slide fluorescent scanners such as those from Akoya Bioscience (previously Perkin Elmer) and Hamamatsu, has made this approach more amenable to high-throughput drug discovery and translational research.

The use of multi-immunofluorescence (mIF) in cell phenotyping has now transitioned to the mainstream and been validated and employed by multiple laboratories. mIF opens up a wide range of analytical approaches for endpoint analysis, and in particular allows assessment of the effects of a therapeutic agent on specific cell types, stratifying outcomes in both tumour and non-tumour regions accordingly. An example of this approach is shown in Fig. 3; an antibody against a tumour marker such as a specific cytokeratin can be used to mask tumour regions. Cell death and proliferation responses can then be separately quantified in tumour and non-tumour areas by using antibodies against markers such as cPARP and Ki67, respectively. This type of analysis allows for the stratification of responders and nonresponders to a given therapy, as shown in Fig. 3 for an experimental compound. Incorporation of antibody staining for a specific pharmacodynamic marker allows validation of on-target effects of the therapy. We are currently using this empirical approach for assessing PDE drug responses in tumour and stroma regions.

**Fig. 4:**
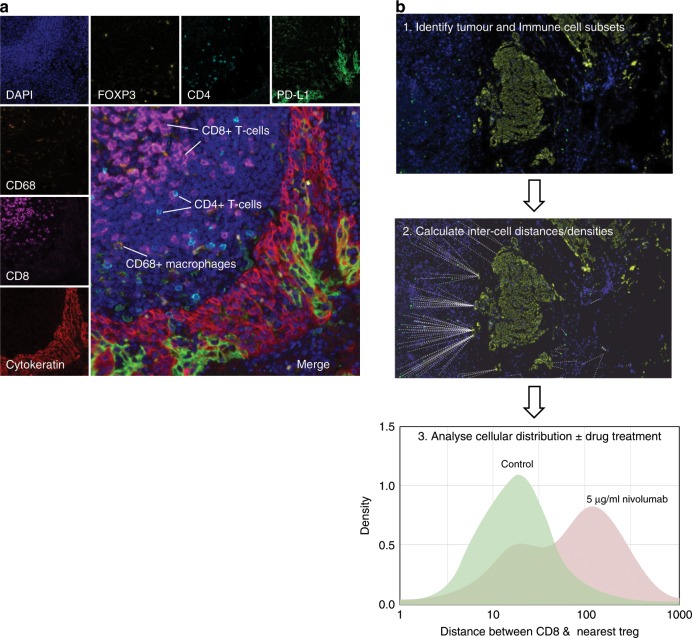
Multiplexed immunofluorescence. **a** A section of a PDE stained with DAPI as well as antibodies specific for immune cell markers (CD4, CD8, CD68 and FOXP3), PD-L1 and pan-cytokeratin followed by TSA-based fluorescent labelling is shown. This method allows for the characterisation of immune-cell subsets in the tumour microenvironment. **b** Measurement of inter-cell distances in PDEs pre- and post drug treatment. In this example, a melanoma PDE is analysed pre- and post-treatment with the anti-PD1 immunotherapy Nivolumab. To analyse inter-cell distances, PDEs are first stained with relevant cell markers such as tumour or immune-cell subsets followed by digital scanning of images (1). Cells are then identified and phenotyped using Inform software, and distances calculated using the R programming environment (2). The example shown in the histogram (3), indicates increased distance between CD8^+^ effector T cells and CD4^+^FOXP3^+^ regulatory T cells (Tregs) following Nivolumab treatment, confirming on-target effects of this anti-PD1 immunotherapy within the PDE. These images are the authors’ unpublished original data.

Immune-cell profiling is particularly important in the context of analysing PDE responses to immunotherapies, and mIF allows for the tracking of multiple cell types simultaneously (Fig. 4a). Parra et al.^94^ used multiple mIF panels to identify immune-cell subsets in NSCLC, and subsequently demonstrated the expression of the immune-checkpoint protein programmed cell death ligand (PD-L1) in macrophages and malignant cells. Similarly, Park et al.^95^ used the same methodology to evaluate whether TILs could be used to predict responsiveness to preoperative chemoradiotherapy in rectal cancers. By co-staining for CD4, CD8, PD-L1, FOXP3 and cytokeratin they were able to correlate TIL density to response.

In addition to raw immune-cell numbers, it is becoming increasingly clear that the localisation of immune-cell subsets in the TME also plays a critical role in drug response. Tumours can be separated into hot (immune infiltrates in the tumour core), cold (showing no immune-cell infiltration) and immune excluded (immune cells aggregating at the tumour boundaries).^96^ Using PDEs, it is possible to investigate whether, for example, following drug treatments, a tumour transitions from an immune-excluded phenotype to that of a ‘hot’ one and if this is predictive of patient drug response. Ongoing work in our laboratory is investigating the use of PDEs for this purpose and for the testing of immunotherapies. Encouraging in this regard is the observation that treatment of melanoma PDEs with the immune-checkpoint inhibitor (ICI) Nivolumab increases the distance between CD8^+^ effector T cells and CD4^+^FOXP3^+^ regulatory T cells, confirming measurable biological effects of this anti-PD1 antibody in PDEs (Fig. 4b).

By using PDEs, it is also possible to investigate drug penetrance using mass spectrometry imaging (MSI) such as matrix-assisted laser desorption/ionisation (MALDI)–MSI. Using this method, we have demonstrated that PDEs from cisplatin-resistant NSCLCs exclude the drug from tumour regions.^79^ Using a similar method, Sørensen et al. were able to study the skin penetrance and distribution of drug compounds in a human skin explant model system.^97^ Processing of PDEs for FFPE also does not preclude genomic or transcriptomic approaches being used for biomarker analysis. Next-generation sequencing of RNA or DNA isolated from FFPE tissue is now routinely performed and validated.^98,99^ Extraction of defined regions within PDEs using laser microdissection^100,101^ would also potentially allow correlation between genomic/transcriptomic heterogeneity and drug responses.

## Advantages and limitations of PDEs

An overview of the major advantages and limitations of the PDE platform is provided in Box 1. The proven predictive nature of drug responses in patients is a significant advantage of the PDE platform compared with other preclinical platforms, particularly 2D cell lines and cell line–mouse xenograft models. Other advantages include the ability to culture the surrounding normal tissue in parallel to assess the on-target specificity of anti-cancer agents, and depending on the endpoint chosen, the potential to generate personalised data rapidly. The generation and culture of PDEs is also relatively inexpensive compared with the production and maintenance of mouse models or the isolation and culture of organoids, which often require costly cell culture reagents and matrices for their maintenance. However, perhaps the most outstanding feature is the fact that PDEs contextually preserve the original architecture of the tissue, thus allowing assessment of responses to agents that target the TME.^69,89^ There has been a desperate search for models that allow the testing and stratification of ICIs, such as monoclonal antibodies that target PD-L1 or its receptor.^16^ These immunotherapies show unprecedented activity in a range of cancer types. However, not all patients respond, and side effects are common. Our preliminary data using PDEs (see Fig. 4b) indicate patient-specific responses to immunotherapies, suggesting that this platform provides a unique opportunity to identify novel biomarkers of response that could be used to stratify patients.

A concern with the PDE platform is that drug diffusion/uptake may be inadequate throughout the sample. Our own work focussed on monitoring the distribution of drugs within samples by using MALDI–MSI and showed that in the case of cisplatin-treated PDEs, platinum ions are distributed throughout samples that respond to the drug, in both tumour and stroma areas.^79^ The fact that PDEs also show biological responses to immunotherapies (Fig. 4b) indicates that antibody-directed therapies also have sufficient access to the PDEs. However, drug diffusion/uptake will vary between different types of anti-cancer agents, whether they be small molecules, antibodies, nucleic acids or proteins, and this needs to be taken into account when considering whether to apply the PDE platform to the testing of a particular agent.

Another limitation of the PDE platform is that PDEs are only retained intact for short periods of time, typically for up to 72 h. Drug treatments need to be undertaken during a window of time between retention of the original tumour architecture and disintegration due to adaptation of the PDE to the culture environment. Analysis of drug responses also must take into account any impact of necrosis that occurs as a result of adaptation to the culture conditions. It is generally recommended that culture conditions are first optimised for any new tumour type under investigation by testing different media, serum and other additives. For example, we have found that the integrity of breast and endometrial tumours is improved if autologous serum is used in the culture media. However, extending the integrity of PDEs for much longer periods needs further research, possibly involving the development of scaffolds or perfused channels to prolong the 3D architecture of the original tumours. Finally, some tumours are too intrinsically necrotic to be amenable to PDE culture (estimated at < 20%),^79^ although this feature would also prohibit PDX and organoid derivation. Overall, while the PDE approach allows assessment of intrinsic sensitivity or resistance of tumours to various anti-cancer agents, the fact that integrity is lost over time using present culture methods prohibits use of the platform for studying the mechanism(s) of acquired drug resistance or invasion and metastasis.

Box 1. Advantages and limitations of patient-derived explant cultures **Advantages**

- Uses patient-relevant material
- Surrounding normal tissue can be studied in parallel
- Patient information available
- Tumour diagnostic information available
- Tumours retain proliferative capacity
- Tissue architecture maintained
- Tumour–stroma interactions maintained
- Models intra-/inter-tumoural heterogeneity
- Agents targeting both tumour and stroma can be assessed
- Platform is predictive of in vivo drug response
- Multiple types of endpoint analysis can be applied (spatial and non-spatial)
- Multiple pharmacodynamic biomarkers can be profiled
- Allows correlation of drug response with tumour pathology/patient characteristics/genetics/drug distribution/biomarker expression
- Relatively inexpensive
- Rapid generation of drug response data

**Limitations**

- Only applicable to surgically resected tumours
- Access to tumours dependent on good links with surgeons and pathologists
- Experimental results affected by tumour integrity
- Tissue architecture disintegrates within short time frames (typically > 3 d)
- Not suitable where in vivo drug metabolism is required
- Poor model for invasion and metastasis
- Difficult to perform high-throughput drug screens
- Not suitable for acquired drug-resistance studies

## Conclusions and future perspectives

In summary, our view is that no preclinical model can fully recapitulate the complexity of tumours within patients or the physiological parameters determining intrinsic and acquired drug responses. Therefore, the best approach is to use a combination of models to gain the most insightful information on tumour drug responses, drug metabolism, pharmacodynamic biomarkers etc. prior to clinical translation.

PDE culture systems have been in existence since the 1950s, but despite strong evidence for their clinical predictivity, they have not been widely incorporated into the cancer drug development pipeline. Here, we have presented strong evidence that PDEs should be resurrected as a powerful preclinical platform for generating proof-of-concept information. By doing so, PDEs would provide a robust rationale for the continuation or discontinuation of a compound, thus reducing the high attrition rate currently observed in anti-cancer drug development.

## Data Availability

Not applicable.

## References

[1] DiMasi JA, Grabowski HG, Hansen RW (2015). The cost of drug development. N. Engl. J. Med..

[2] Seruga B, Ocana A, Amir E, Tannock IF (2015). Failures in phase III: causes and consequences. Clin. Cancer Res..

[3] Kola I, Landis J (2004). Can the pharmaceutical industry reduce attrition rates?. Nat. Rev. Drug Discov..

[4] Moreno L, Pearson AD (2013). How can attrition rates be reduced in cancer drug discovery?. Expert Opin. Drug Discov..

[5] Paul SM, Mytelka DS, Dunwiddie CT, Persinger CC, Munos BH, Lindborg SR (2010). How to improve R&D productivity: the pharmaceutical industry’s grand challenge. Nat. Rev. Drug Discov..

[6] Ocana A, Tannock IF (2011). When are “positive” clinical trials in oncology truly positive?. J. Natl Cancer Inst..

[7] Scannell JW, Blanckley A, Boldon H, Warrington B (2012). Diagnosing the decline in pharmaceutical R&D efficiency. Nat. Rev. Drug Discov..

[8] Ocana A, Amir E, Vera-Badillo F, Seruga B, Tannock IF (2013). Phase III trials of targeted anticancer therapies: redesigning the concept. Clin. Cancer Res..

[9] Booth B, Glassman R, Ma P (2003). Oncology’s trials. Nat. Rev. Drug Discov..

[10] Gerlinger M, Rowan AJ, Horswell S, Math M, Larkin J, Endesfelder D (2012). Intratumor heterogeneity and branched evolution revealed by multiregion sequencing. N. Engl. J. Med..

[11] Jamal-Hanjani M, Wilson GA, McGranahan N, Birkbak NJ, Watkins TBK, Veeriah S (2017). Tracking the evolution of non-small-cell lung cancer. N. Engl. J. Med..

[12] Kanzaki R, Naito H, Kise K, Takara K, Eino D, Minami M (2017). Gas6 derived from cancer-associated fibroblasts promotes migration of Axl-expressing lung cancer cells during chemotherapy. Sci. Rep..

[13] Arlauckas, S. P., Garris, C. S., Kohler, R. H., Kitaoka, M., Cuccarese, M. F., Yang, K. S. et al. In vivo imaging reveals a tumor-associated macrophage-mediated resistance pathway in anti-PD-1 therapy. *Sci. Transl. Med*. **9**, eaal3604 (2017). 10.1126/scitranslmed.aal3604 PMC5734617 28490665

[14] Olson B, Li Y, Lin Y, Liu ET, Patnaik A (2018). Mouse models for cancer immunotherapy research. Cancer Discov..

[15] Zitvogel L, Pitt JM, Daillere R, Smyth MJ, Kroemer G (2016). Mouse models in oncoimmunology. Nat. Rev. Cancer.

[16] Bertolini F (2019). Desperately seeking…models to find the right partner and the best use for checkpoint inhibitors. Br. J. Cancer.

[17] Unger C, Kramer N, Walzl A, Scherzer M, Hengstschlager M, Dolznig H (2014). Modeling human carcinomas: physiologically relevant 3D models to improve anti-cancer drug development. Adv. Drug Deliv. Rev..

[18] Sharma SV, Haber DA, Settleman J (2010). Cell line-based platforms to evaluate the therapeutic efficacy of candidate anticancer agents. Nat. Rev. Cancer.

[19] Yang W, Soares J, Greninger P, Edelman EJ, Lightfoot H, Forbes S (2013). Genomics of drug sensitivity in cancer (GDSC): a resource for therapeutic biomarker discovery in cancer cells. Nucleic Acids Res..

[20] Garnett MJ, Edelman EJ, Heidorn SJ, Greenman CD, Dastur A, Lau KW (2012). Systematic identification of genomic markers of drug sensitivity in cancer cells. Nature.

[21] Ghandi M, Huang FW, Jane-Valbuena J, Kryukov GV, Lo CC, McDonald ER (2019). Next-generation characterization of the cancer cell line encyclopedia. Nature.

[22] Drost J, Clevers H (2018). Organoids in cancer research. Nat. Rev. Cancer.

[23] van de Wetering M, Francies HE, Francis JM, Bounova G, Iorio F, Pronk A (2015). Prospective derivation of a living organoid biobank of colorectal cancer patients. Cell.

[24] Pauli C, Hopkins BD, Prandi D, Shaw R, Fedrizzi T, Sboner A (2017). Personalized in vitro and in vivo cancer models to guide precision medicine. Cancer Discov..

[25] Weeber F, van de Wetering M, Hoogstraat M, Dijkstra KK, Krijgsman O, Kuilman T (2015). Preserved genetic diversity in organoids cultured from biopsies of human colorectal cancer metastases. Proc. Natl Acad. Sci. USA.

[26] Fujii M, Shimokawa M, Date S, Takano A, Matano M, Nanki K (2016). A colorectal tumor organoid library demonstrates progressive loss of niche factor requirements during tumorigenesis. Cell Stem Cell.

[27] Vlachogiannis G, Hedayat S, Vatsiou A, Jamin Y, Fernandez-Mateos J, Khan K (2018). Patient-derived organoids model treatment response of metastatic gastrointestinal cancers. Science.

[28] Sontheimer-Phelps, A., Hassell, B. A. & Ingber, D. E. Modelling cancer in microfluidic human organs-on-chips. *Nat. Rev. Cancer* **19**, 65–81 (2019). 10.1038/s41568-018-0104-6 30647431

[29] Calibasi Kocal G, Guven S, Foygel K, Goldman A, Chen P, Sengupta S (2016). Dynamic microenvironment induces phenotypic plasticity of esophageal cancer cells under flow. Sci. Rep..

[30] Gjorevski N, Sachs N, Manfrin A, Giger S, Bragina ME, Ordonez-Moran P (2016). Designer matrices for intestinal stem cell and organoid culture. Nature.

[31] Ohlund D, Handly-Santana A, Biffi G, Elyada E, Almeida AS, Ponz-Sarvise M (2017). Distinct populations of inflammatory fibroblasts and myofibroblasts in pancreatic cancer. J. Exp. Med..

[32] Seino T, Kawasaki S, Shimokawa M, Tamagawa H, Toshimitsu K, Fujii M (2018). Human pancreatic tumor organoids reveal loss of stem cell niche factor dependence during disease progression. Cell Stem Cell.

[33] Gao D, Vela I, Sboner A, Iaquinta PJ, Karthaus WR, Gopalan A (2014). Organoid cultures derived from patients with advanced prostate cancer. Cell.

[34] Sachs N, de Ligt J, Kopper O, Gogola E, Bounova G, Weeber F (2018). A living biobank of breast cancer organoids captures disease heterogeneity. Cell.

[35] Gould SE, Junttila MR, de Sauvage FJ (2015). Translational value of mouse models in oncology drug development. Nat. Med..

[36] Gengenbacher N, Singhal M, Augustin HG (2017). Preclinical mouse solid tumour models: status quo, challenges and perspectives. Nat. Rev. Cancer.

[37] Hodgkinson CL, Morrow CJ, Li Y, Metcalf RL, Rothwell DG, Trapani F (2014). Tumorigenicity and genetic profiling of circulating tumor cells in small-cell lung cancer. Nat. Med..

[38] Ireson, C. R., Alavijeh, M. S., Palmer, A. M., Fowler, E. R. & Jones, H. J. The role of mouse tumour models in the discovery and development of anticancer drugs. *Br. J. Cancer* **121**, 101–108 (2019). 10.1038/s41416-019-0495-5 PMC6738037 31231121

[39] Hidalgo M, Amant F, Biankin AV, Budinska E, Byrne AT, Caldas C (2014). Patient-derived xenograft models: an emerging platform for translational cancer research. Cancer Discov.

[40] Tentler JJ, Tan AC, Weekes CD, Jimeno A, Leong S, Pitts TM (2012). Patient-derived tumour xenografts as models for oncology drug development. Nat. Rev. Clin. Oncol..

[41] DeRose YS, Wang G, Lin YC, Bernard PS, Buys SS, Ebbert MT (2011). Tumor grafts derived from women with breast cancer authentically reflect tumor pathology, growth, metastasis and disease outcomes. Nat. Med..

[42] Eirew P, Steif A, Khattra J, Ha G, Yap D, Farahani H (2015). Dynamics of genomic clones in breast cancer patient xenografts at single-cell resolution. Nature.

[43] Bruna A, Rueda OM, Greenwood W, Batra AS, Callari M, Batra RN (2016). A biobank of breast cancer explants with preserved intra-tumor heterogeneity to screen anticancer compounds. Cell.

[44] Hidalgo M, Bruckheimer E, Rajeshkumar NV, Garrido-Laguna I, De Oliveira E, Rubio-Viqueira B (2011). A pilot clinical study of treatment guided by personalized tumorgrafts in patients with advanced cancer. Mol. Cancer Ther..

[45] Izumchenko E, Paz K, Ciznadija D, Sloma I, Katz A, Vasquez-Dunddel D (2017). Patient-derived xenografts effectively capture responses to oncology therapy in a heterogeneous cohort of patients with solid tumors. Ann. Oncol..

[46] Lai Y, Wei X, Lin S, Qin L, Cheng L, Li P (2017). Current status and perspectives of patient-derived xenograft models in cancer research. J. Hematol. Oncol..

[47] Drake AC, Chen Q, Chen J (2012). Engineering humanized mice for improved hematopoietic reconstitution. Cell. Mol. Immunol..

[48] Wang M, Yao LC, Cheng M, Cai D, Martinek J, Pan CX (2018). Humanized mice in studying efficacy and mechanisms of PD-1-targeted cancer immunotherapy. FASEB J.

[49] King MA, Covassin L, Brehm MA, Racki W, Pearson T, Leif J (2009). Human peripheral blood leucocyte non-obese diabetic-severe combined immunodeficiency interleukin-2 receptor gamma chain gene mouse model of xenogeneic graft-versus-host-like disease and the role of host major histocompatibility complex. Clin. Exp. Immunol..

[50] Jangalwe S, Shultz LD, Mathew A, Brehm MA (2016). Improved B cell development in humanized NOD-scid IL2Rgamma(null) mice transgenically expressing human stem cell factor, granulocyte-macrophage colony-stimulating factor and interleukin-3. Immun. Inflamm. Dis..

[51] Saito Y, Ellegast JM, Rafiei A, Song Y, Kull D, Heikenwalder M (2016). Peripheral blood CD34(+) cells efficiently engraft human cytokine knock-in mice. Blood.

[52] Leighton J (1951). A sponge matrix method for tissue culture; formation of organized aggregates of cells in vitro. J. Natl Cancer Inst..

[53] Hoffman RM (2018). 3D sponge-matrix histoculture: an overview. Methods Mol. Biol..

[54] Leighton J (1954). The growth patterns of some transplantable animal tumors in sponge matrix tissue culture. J. Natl Cancer Inst..

[55] Leighton J, Kline I, Belkin M, Legallais F, Orr HC (1957). The similarity in histologic appearance of some human cancer and normal cell strains in sponge-matrix tissue culture. Cancer Res..

[56] Freeman AE, Hoffman RM (1986). In vivo-like growth of human tumors in vitro. Proc. Natl Acad. Sci. USA.

[57] Vescio RA, Redfern CH, Nelson TJ, Ugoretz S, Stern PH, Hoffman RM (1987). In vivo-like drug responses of human tumors growing in three-dimensional gel-supported primary culture. Proc. Natl Acad. Sci. USA.

[58] Vescio RA, Connors KM, Kubota T, Hoffman RM (1991). Correlation of histology and drug response of human tumors grown in native-state three-dimensional histoculture and in nude mice. Proc. Natl Acad. Sci. USA.

[59] Furukawa T, Kubota T, Hoffman RM (1995). Clinical applications of the histoculture drug response assay. Clin. Cancer Res..

[60] Yoshimasu T, Oura S, Hirai I, Kokawa Y, Sasaki R, Honda K (2003). Histoculture drug response assay (HDRA) guided induction concurrent chemoradiotherapy for mediastinal node-positive non-small cell lung cancer. Gan To Kagaku Ryoho.

[61] Yoshimasu, T., Oura, S., Hirai, I., Kokawa, Y., Hata, K., Kawago, M. et al. Cut-off level of docetaxel, paclitaxel and gemcitabine in histoculture drug response assay for non-small cell lung cancer. *Gan To Kagaku Ryoho* **32**, 1013–1016 (2005). 16044964

[62] Yoshimasu T, Oura S, Hirai I, Tamaki T, Kokawa Y, Hata K (2007). Data acquisition for the histoculture drug response assay in lung cancer. J. Thorac. Cardiovasc. Surg..

[63] Tanahashi M, Yamada T, Moriyama S, Suzuki E, Niwa H (2008). The effect of the histoculture drug response assay (HDRA) based perioperative chemotherapy for non-small cell lung cancer. Kyobu Geka.

[64] Hayashi Y, Kuriyama H, Umezu H, Tanaka J, Yoshimasu T, Furukawa T (2009). Class III beta-tubulin expression in tumor cells is correlated with resistance to docetaxel in patients with completely resected non-small-cell lung cancer. Intern. Med..

[65] Centenera MM, Raj GV, Knudsen KE, Tilley WD, Butler LM (2013). Ex vivo culture of human prostate tissue and drug development. Nat. Rev. Urol..

[66] Centenera MM, Gillis JL, Hanson AR, Jindal S, Taylor RA, Risbridger GP (2012). Evidence for efficacy of new Hsp90 inhibitors revealed by ex vivo culture of human prostate tumors. Clin. Cancer Res..

[67] Dean JL, McClendon AK, Hickey TE, Butler LM, Tilley WD, Witkiewicz AK (2012). Therapeutic response to CDK4/6 inhibition in breast cancer defined by ex vivo analyses of human tumors. Cell Cycle.

[68] Pirnia F, Frese S, Gloor B, Hotz MA, Luethi A, Gugger M (2006). Ex vivo assessment of chemotherapy-induced apoptosis and associated molecular changes in patient tumor samples. Anticancer Res..

[69] Mariel GC, Edith CI, Pilar CR, Elena GN, Humberto RM, Guadalupe MM (2018). Expression of NK cell surface receptors in breast cancer tissue as predictors of resistance to antineoplastic treatment. Technol. Cancer Res. Treat..

[70] Maund SL, Nolley R, Peehl DM (2014). Optimization and comprehensive characterization of a faithful tissue culture model of the benign and malignant human prostate. Lab. Invest..

[71] Yoshimasu T, Ohta F, Oura S, Tamaki T, Shimizu Y, Naito K (2009). Histoculture drug response assay for gefitinib in non-small-cell lung cancer. Gen. Thorac. Cardiovasc. Surg..

[72] Colangelo D, Guo HY, Connors KM, Kubota T, Silvestro L, Hoffman RM (1992). Correlation of drug response in human tumors histocultured in vitro with an image-analysis MTT end point and in vivo xenografted in nude mice. Anticancer Res.

[73] Vaira V, Fedele G, Pyne S, Fasoli E, Zadra G, Bailey D (2010). Preclinical model of organotypic culture for pharmacodynamic profiling of human tumors. Proc. Natl Acad. Sci. USA.

[74] Wei B, Wang J, Zhang X, Qian Z, Wu J, Sun Y (2017). Combination of histoculture drug response assay and qPCR as an effective method to screen biomarkers for personalized chemotherapy in esophageal cancer. Oncol. Lett..

[75] Majumder B, Baraneedharan U, Thiyagarajan S, Radhakrishnan P, Narasimhan H, Dhandapani M (2015). Predicting clinical response to anticancer drugs using an ex vivo platform that captures tumour heterogeneity. Nat. Commun..

[76] Goldman A, Majumder B, Dhawan A, Ravi S, Goldman D, Kohandel M (2015). Temporally sequenced anticancer drugs overcome adaptive resistance by targeting a vulnerable chemotherapy-induced phenotypic transition. Nat. Commun..

[77] Bhattacharyya S, Sekar V, Majumder B, Mehrotra DG, Banerjee S, Bhowmick AK (2017). CDKN2A-p53 mediated antitumor effect of Lupeol in head and neck cancer. Cell Oncol..

[78] Brijwani N, Jain M, Dhandapani M, Zahed F, Mukhopadhyay P, Biswas M (2017). Rationally co-targeting divergent pathways in KRAS wild-type colorectal cancers by CANscript technology reveals tumor dependence on Notch and Erbb2. Sci. Rep..

[79] Karekla E, Liao WJ, Sharp B, Pugh J, Reid H, Quesne JL (2017). Ex vivo explant cultures of non-small cell lung carcinoma enable evaluation of primary tumor responses to anticancer therapy. Cancer Res..

[80] Twiddy, D., Naik, S., Mistry, R., Edwards, J., Walker, R. A., Cohen, G. M. et al. A TRAILR 1-specific ligand in combination with doxorubin selectively targets primary breast tumour cells for apoptosis. *Breast Cancer Res*. **12**, 17–18 (2010).

[81] Cai H, Scott E, Kholghi A, Andreadi C, Rufini A, Karmokar A (2015). Cancer chemoprevention: Evidence of a nonlinear dose response for the protective effects of resveratrol in humans and mice. Sci. Transl. Med..

[82] Busacca S, Law EW, Powley IR, Proia DA, Sequeira M, Le Quesne J (2016). Resistance to HSP90 inhibition involving loss of MCL1 addiction. Oncogene.

[83] Kolluri, K. K., Alifrangis, C., Kumar, N., Ishii, Y., Price, S., Michaut, M. et al. Loss of functional BAP1 augments sensitivity to TRAIL in cancer cells. *eLife* **7**, e30224 (2018). 10.7554/eLife.30224 PMC5773178 29345617

[84] Dyer MJ, MacFarlane M, Cohen GM (2007). Barriers to effective TRAIL-targeted therapy of malignancy. J. Clin. Oncol..

[85] Centenera MM, Hickey TE, Jindal S, Ryan NK, Ravindranathan P, Mohammed H (2018). A patient-derived explant (PDE) model of hormone-dependent cancer. Mol. Oncol..

[86] Schiewer MJ, Goodwin JF, Han S, Brenner JC, Augello MA, Dean JL (2012). Dual roles of PARP-1 promote cancer growth and progression. Cancer Discov..

[87] Mohammed H, Russell IA, Stark R, Rueda OM, Hickey TE, Tarulli GA (2015). Progesterone receptor modulates ERalpha action in breast cancer. Nature.

[88] Nguyen EV, Centenera MM, Moldovan M, Das R, Irani S, Vincent AD (2018). Identification of novel response and predictive biomarkers to Hsp90 inhibitors through proteomic profiling of patient-derived prostate tumor explants. Mol. Cell. Proteomics.

[89] Shafi AA, Schiewer MJ, de Leeuw R, Dylgjeri E, McCue PA, Shah N (2018). Patient-derived models reveal impact of the tumor microenvironment on therapeutic response. Eur. Urol. Oncol..

[90] Bobrow MN, Harris TD, Shaughnessy KJ, Litt GJ (1989). Catalyzed reporter deposition, a novel method of signal amplification. Application to immunoassays. J. Immunol. Methods.

[91] Hunyady B, Krempels K, Harta G, Mezey E (1996). Immunohistochemical signal amplification by catalyzed reporter deposition and its application in double immunostaining. J. Histochem. Cytochem..

[92] Stack EC, Wang C, Roman KA, Hoyt CC (2014). Multiplexed immunohistochemistry, imaging, and quantitation: a review, with an assessment of Tyramide signal amplification, multispectral imaging and multiplex analysis. Methods.

[93] Toth ZE, Mezey E (2007). Simultaneous visualization of multiple antigens with tyramide signal amplification using antibodies from the same species. J. Histochem. Cytochem..

[94] Parra ER, Uraoka N, Jiang M, Cook P, Gibbons D, Forget MA (2017). Validation of multiplex immunofluorescence panels using multispectral microscopy for immune-profiling of formalin-fixed and paraffin-embedded human tumor tissues. Sci. Rep..

[95] Park IJ, An S, Kim SY, Lim HM, Hong SM, Kim MJ (2017). Prediction of radio-responsiveness with immune-profiling in patients with rectal cancer. Oncotarget.

[96] Kather, J. N., Suarez-Carmona, M., Charoentong, P., Weis, C. A., Hirsch, D., Bankhead, P. et al. Topography of cancer-associated immune cells in human solid tumors. *eLife* **7**, e36967 (2018). 10.7554/eLife.36967 PMC6133554 30179157

[97] Sorensen IS, Janfelt C, Nielsen MMB, Mortensen RW, Knudsen NO, Eriksson AH (2017). Combination of MALDI-MSI and cassette dosing for evaluation of drug distribution in human skin explant. Anal. Bioanal. Chem..

[98] Oh E, Choi YL, Kwon MJ, Kim RN, Kim YJ, Song JY (2015). Comparison of accuracy of whole-exome sequencing with formalin-fixed paraffin-embedded and fresh frozen tissue samples. PLoS One.

[99] Robbe P, Popitsch N, Knight SJL, Antoniou P, Becq J, He M (2018). Clinical whole-genome sequencing from routine formalin-fixed, paraffin-embedded specimens: pilot study for the 100,000 Genomes Project. Genet. Med..

[100] Bonner RF, Emmert-Buck M, Cole K, Pohida T, Chuaqui R, Goldstein S (1997). Laser capture microdissection: molecular analysis of tissue. Science.

[101] Burgemeister R (2011). Laser capture microdissection of FFPE tissue sections bridging the gap between microscopy and molecular analysis. Methods Mol. Biol..

